# Effect of Intensive Glycemic Control on Myocardial Infarction Outcome in Patients with Type 2 Diabetes Mellitus: A Systematic Review and Meta-Analysis

**DOI:** 10.1155/2023/8818502

**Published:** 2023-02-24

**Authors:** Jiading He, Yangbo Xi, Hingcheung Lam, Keyi Du, Dongping Chen, Zhihui Dong, Jianmin Xiao

**Affiliations:** ^1^Department of Cardiology, The Dongguan Affiliated Hospital of Jinan University, Binhaiwan Central Hospital of Dongguan, Dongguan, China; ^2^Department of The First Clinical Medical College, Jinan University, Guangzhou, China; ^3^Central Laboratory, The Dongguan Affiliated Hospital of Jinan University, Binhaiwan Central Hospital of Dongguan, Dongguan, China

## Abstract

**Background:**

The effect of intensive glucose-lowering treatment on the risk of cardiovascular events in type 2 diabetes remains uncertain, especially the effect on the occurrence of myocardial infarction in patients with type 2 diabetes is still unclear. The purpose of this study was to conduct a systematic review and meta-analysis of relevant RCTs.

**Methods:**

We performed a systematic review of randomized clinical trials (RCTS) and observational studies relevant to this study question. We searched the PubMed and Cochrane databases until June 2022.

**Results:**

We included data on 14 RCTs and 144,334 patients, all of whom had type 2 diabetes. When all studies were considered, intensive glucose-lowering treatment significantly reduced the incidence of MI compared with conventional therapy and the total OR value is 0.90 (CI 0.84, 0.97; *P* = 0.004) when considering all the studies. When the target value of intensive glucose-lowering treatment was considered as HbA1c decrease of more than 0.5%, there was no significant protective effect on MI, the total OR value is 0.88 (CI 0.81, 0.96; *P* = 0.003). When considering all available RCTS, the intensive glucose-lowering treatment group had a protective effect for MACE compared to the conventional treatment group, and the total OR value is 0.92 (CI 0.88, 0.96; *P* < 0.00001). In the available RCTs, for the patients with a history of prior CAD, the total OR value is 0.94 (CI 0.89, 0.99; *P* = 0.002). And there was no difference in the incidence of hypoglycemic events between the intensive and conservative treatment groups.

**Conclusion:**

Our data support the positive protective effect of glucose-lowering therapy on MI in patients with T2DM, but there is no significant effect of intensive glucose-lowering. In addition, we found no greater protective effect of enhanced glucose control in the HbA1c reduction of more than 0.5%, and no difference in the incidence of adverse events compared with the HbA1c reduction of less than 0.5%.

## 1. Background

The incidence of type 2 diabetes (T2D) is increasing in all regions of the world. T2D is commonly associated with cardiovascular disease (CVD), such as atherosclerosis, acute myocardial infarction, and chronic kidney disease. Recently, more and more studies have found that the degree of T2D control is associated with the occurrence of cardiovascular events. Previous large clinical trials, including VADT [1], ACCORD [2], and ADVANCE [3], did not prove that glycemic control significantly reduced the incidence of cardiovascular events compared with the standard group. However, some studies, such as the DCCT/EDIC [4] and UKPDS [5] cohorts, have shown that intensive glycemic control has a long-term protective effect on the occurrence of cardiovascular events, even after intensive therapy has been discontinued. With the emergence of new drugs such as SGLT2 inhibitors and the GLP-1 receptor agonist, for T2D patients with the risk of cardiovascular events, the EMPA-REG OUTCOME [6] and CANVAS [7] has been mentioned that SGTLT2 inhibitors and the GLP-1 receptor agonist have protection effects on the incidence and mortality of cardiovascular events. There is still a lot of controversy over whether intensive glucose-lowering treatment should be carried out in the T2D patients with cardiovascular risk factors.

Recent studies have also confirmed that hyperglycemia is associated with acute myocardial infarction (AMI) inflammatory indicators and infarct size to some extent [8]. The mediation of hyperglycemia may not only amplify the process of inflammatory response but in turn, insulin resistance caused by inflammation also promotes the occurrence of hyperglycemia, triggering a series of vicious cycles, which may eventually lead to increased load and rupture of atherosclerotic plaques in coronary arteries, thus increasing the incidence of MACE or myocardial infarction [8, 9]. Interestingly, the clinical status of hyperglycemia on admission was also characterized by overall hemodynamic instability, with higher heart rate, higher Killip grading, and a higher prevalence of atrial fibrillation. Hyperglycemia improves the activity of the inflammatory and sympathetic nervous system, worsens endothelial function, and may produce more abundant reactive oxygen species and proinflammatory/procoagulant markers. Therefore, this result may not only lead to coronary artery spasm and nonreflow phenomenon, but also may lead to myocardial membrane damage and calcium overload, thus increasing arrhythmia load and reducing myocardial contractile force. The short-term and long-term prognosis of AMI patients was poor [10–12].

Existing meta-analyses of the effect of intensive glycemic control versus conservative treatment on cardiovascular events are missing from recently published studies. In addition, most of the studies did not screen according to the degree of glycemic enhancement, and the selected studies had different glycemic control goals, leading to different results on the occurrence of cardiovascular events.

The effect of intensive glucose-control on cardiovascular events, especially myocardial infarction, remains controversial [13]. The degree of blood glucose decline is also controversial in relation to the incidence of myocardial infarction [14]. Therefore, we systematically reviewed the literature and selected all available randomized clinical trials (RCTS) to answer this specific research question: Does intensive glycemic control provide benefits for the occurrence of cardiovascular events, particularly myocardial infarction? In addition, as far as we know, whether the degree of glycemic control has an impact on cardiovascular events, especially myocardial infarction, has not been clearly studied. Moreover, we analyzed the influence of previous cardiovascular events on MACE by subgroup.

## 2. Methods

### 2.1. Literature Search and Research Selection

We searched the Pubmed and Cochrane until June 2022. Only relevant English literature is collected. We use the terms “Myocardial Infarction [Mesh Terms]”, “Acute Coronary Syndrome [Mesh Terms]”, “Diabetes Mellitus, Type 2[MeshTerms]”, “Glycemic Control [MeshTerms]”, “Cardiovascular Diseases[Mesh Terms]”, “Cardiovascular death[All Fields]”, “Coronary Heart Disease[All Fields]”, “Strokes[All Fields]”, “Major adverse cardiovascular events[All Fields]”, “Cardiovascular mortality[All Fields]”, “Intensive glucose control[All Fields]”, “Type 2 Diabetes[All Fields]”, and “Blood Glucose Control[All Fields]” as key terms.

The applicable criteria for RCTS are the following: (a) all patients with type 2 diabetes; (b) must include intensive glucose-control treatment with a conventional treatment (either standard diabetes care, low-standard glycemic treatment, or placebo); (c) the magnitude of glucose reduction must be clearly targeted (either a prespecified HbA1c or fasting glucose target, or defined in terms of intensive glucose-control treatment); (d) follow-up outcomes must include data on the incidence of myocardial infarction, or major adverse cardiovascular events (MACE, defined as the sum of cardiovascular death, myocardial infarction, and stroke) were reported as one of the endpoints; and (e) postactivity and observational follow-up were included, which was consistent with the proposed research questions. Exclusion criteria were as follows: (a) the trial did not include patients with type 2 diabetes or included patients with type 1 diabetes; (b) there is no preset blood glucose standard, or there is no specific intensive glucose-control drugs; (c) the trial consists of multiple interventions; (d) the trial did not contain data on the outcome of MI or on the outcome of MACE, and no secondary endpoints were collected; (e) the trials not having a postactive, observational phase; and (f) trials performed with drugs no longer on the market due to unacceptable toxicity.

### 2.2. Data Extraction and Research Quality Assessment

We used preprepared standardized forms to extract and collect data from selected randomized controlled trials and studies. Included are the study design, follow-up time, treatment intervention, duration of diabetes at baseline, initial treatment of Hba1c, posttreatment of HBA1C, number of patients in the control group and the experimental group, number of MACE, and number of myocardial infarction. The Jadad score was used independently to assess the quality of the selected RCTS, with the result that the selected studies were double-blind randomized placebo-controlled trials and thus scored over 3 points [15]. And the methodological quality of the included trials was also judged using the Cochrane Collaboration's tool for assessing the risk of bias [16] and the Grading of Recommendations Assessment, Development and Evaluation (GRADE) system for rating the quality of evidence [17].

### 2.3. Statistical Analysis

In this meta-analysis, aggregated data were used, and a quantitative synthesis of the findings from the included studies was performed. Because all adverse outcomes were binary indicators, the number of events and the number of patients in each group were used to calculate log odds ratios (OR) with 95% confidence intervals for every RCT. We assessed the potential heterogeneity of mortality across studies using statistical data and *I* measures, where an *I* of 75% or higher was considered to represent high heterogeneity [18]. Heterogeneity between subgroups was assessed based on the type of treatment between subgroups. We also analyzed cooccurring cardiovascular risk factors between the study groups to assess the consistency of myocardial infarction treatment outcomes and CV death endpoints. *P* values of both sides were calculated; *P* < 0.05 was considered to be significant for combined OR results. *P* < 0.01 was considered to be an important factor in subgroup interaction to compensate for multiple tests. Statistical heterogeneity between trials was assessed using *I*^2^ statistics. The significance of heterogeneity was set as *I*^2^ < 50% or *P* < 0.1. In this case, the random effects model is used for analysis [18]. When *P* < 0.1 and *I*^2^ < 50%, heterogeneity was considered insignificant. The heterogeneity of treatment effects among studies was assessed using both Cochran's Q and Higgins's *I*^2^ statistics [16]. Publication bias was detected using funnel plots and Egger's regression asymmetry test [19]. Statistical analysis was performed using Review Manager 5.

## 3. Result

### 3.1. Inclusion Flow and Exclusion Studies

Inclusion flow is depicted in Figure S1. Of the 154 articles identified in the literature search, 53 articles were excluded after abstracts or full-text reviews, and 49 articles were excluded. Among them, 13 studies were excluded because of no pertinent design, and 3 studies were excluded because of no follow-up data [20–22]. And the HI-5 study [23], the DIGAMI study [24], and other 2 articles were excluded due to short-term intensive treatment. And there are 2 articles were excluded due to multifactorial intervention. And the DIGAMI 1 study [25], the DIGAMI 2 study [26], the RECORD study [27], and the VADT study (2015) [28] were excluded due to fewer than 1000 patients follow-up. And the DAPA-HF study [29], the AleCardio study [30], the ACE study [31], and the other 1 articles were excluded because their patients with Type 2 diabetes were not identified. And the VERTIS CV study [32], the SUSTAIN 4 study [33], the CAROLINA study [34], the DURATION-1 study [35], and other 3 articles were excluded because no MACE was followed up. And the HEART2D study [36] and other 1 articles were excluded because of no intensive glucose-lowering treatment.

### 3.2. Included Studies and Patients' Characteristics

Overall, we eventually included 14 RCT manuscripts [1–3, 6, 7, 37–45] and included data on 144,334 patients, all of whom had type 2 diabetes. Their mean age was 63.8 years, the mean duration of diabetes fluctuated between 7.2 and 14.75 years, and the mean follow-up period fluctuated between 1.5 and 5.4 years. The main features of the observational studies included are shown in Supplementary Table 1. At 1 and 2 years after diagnosis, patients were further classified for glycemic control based on a decrease of more than 0.5% in HbA1c compared with the conservative treatment group. These studies had a low or unclear risk of bias for seven domains of potential risk of bias, except the VADT study suspected selection bias (Figures S2 and S3). No clear evidence of publication bias was noted for all outcomes by funnel plot and/or Egger's test (all *P* > 0.09) (Figure S4).

### 3.3. Effects of Intensice Glucose Control and Conventional Glucose Control on MI under Different Levels of Blood Glucose Control

#### 3.3.1. Considering All the Available RCTs

**Figure 1:**
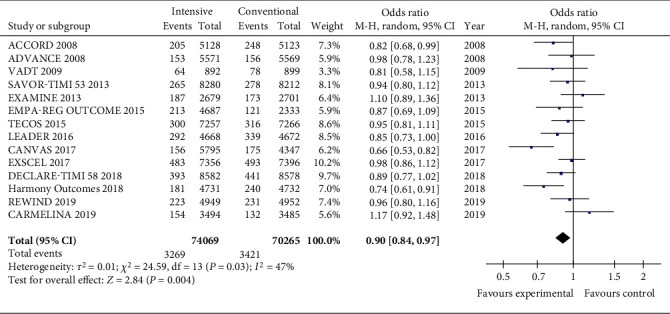
Forest plots of the meta-analyses of the effect of intensive glucose-lowering treatment on MI incidence considering all available data.

When all studies were considered, intensive glucose-lowering treatment significantly reduced the incidence of MI compared with conventional therapy (Figure 1). The total OR value is 0.90 (CI 0.84, 0.97; *P* = 0.004) when considering all the studies. Heterogeneity was not significant when all studies were included (*I*^2^ = 47%, *P* = 0.03).

#### 3.3.2. Considering RCTs in which HbA1c Decreased by More than 0.5%

**Figure 2:**
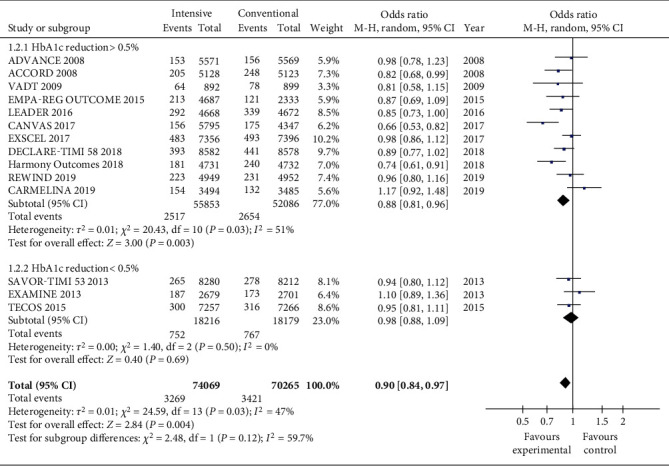
Forest plots of the meta-analyses of the effect of two groups of different HbA1c reductions on MI incidence considering all available data.

When the target value of intensive glucose-lowering treatment was considered as an HbA1c decrease of more than 0.5%, the experimental group was more protective, but there was no significant protective effect on MI (Figure 2). When HbA1c dropped by more than 0.5%, the total OR value is 0.88 (CI 0.81, 0.96; *P* = 0.003), and when HbA1c dropped by less than 0.5%, the total OR value is 0.98 (CI 0.88, 1.09; *P* = 0.69). The variance was not high in both groups (*I*^2^ = 51, *P* = 0.03 decreased by more than 0.5% for HbA1c; *I*^2^ = 0%, *P* = 0.50 for HbA1c reduction less than 0.5%). But the overall variation was considered to be high (*I*^2^ = 59.7%, *P* = 0.12).

### 3.4. Effects of Intensive Glucose Control and Conventional Glucose Control on MACE under Different Levels of Blood Glucose Control

#### 3.4.1. Considering All the Available RCTs

**Figure 3:**
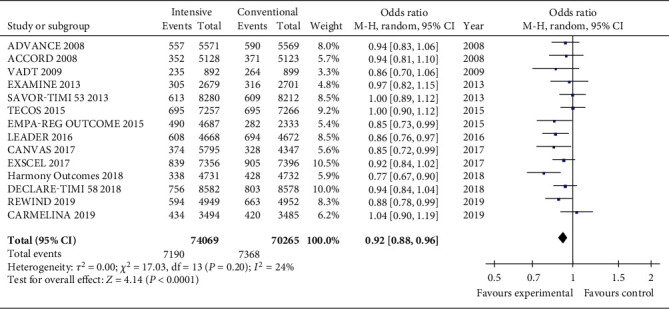
Forest plots of the meta-analyses of the effect of intensive glucose-lowering treatment on MACE incidence considering all available data.

When considering all available RCTs, the intensive glucose-lowering treatment group had a better protective effect for MACE compared to the conventional treatment group (Figure 3). The total OR value is 0.92 (CI 0.88, 0.96; *P* < 0.00001) when considering all the studies. Heterogeneity was not significant when all studies were included (*I*^2^ = 24%, *P* = 0.20).

#### 3.4.2. Considering RCTs in which HbA1c Decreased by More than 0.5%

**Figure 4:**
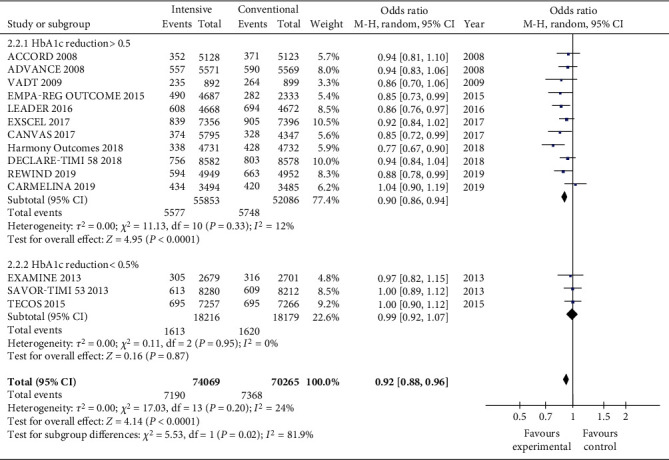
Forest plots of the meta-analyses of the effect of intensive glucose-lowering treatment on MACE incidence considering all available data.

When HbA1c decreased by more than 0.5%, MACE had a significantly more positive effect than those whose HbA1c decreased by <0.5% (Figure 4). The total OR value is 0.90 (CI 0.86, 0.94; *P* < 0.0001) when HbA1c reduction is more than 0.5%, and the total OR value is 0.99 (CI 0.92, 1.07; *P* = 0.87). The variance was not high in both subgroups (*I*^2^ = 12%, *P* = 0.33 decreased by more than 0.5% for HbA1c; *I*^2^ = 0%, *P* = 0.95 for HbA1c reduction less than 0.5%). But the overall variation was considered to be high (*I*^2^ = 81.9%, *P* = 0.02).

#### 3.4.3. Effect of Intensive Glucose-Lowering Treatment on MACE in Patients with a History of Prior CAD

**Figure 5:**
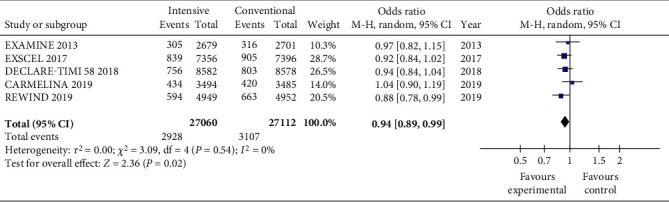
Forest plot for a meta-analysis of the effects of intensive glucose-lowering treatment on the incidence of MACE in patients with a history of prior CAD, taking into account all available data.

If the patient's CAD history was also mentioned in the study, we will collect it. Finally, we used available data to tease out the effect of intensive glucose-lowering treatment on MACE in these patients (Figure 5). It can be seen that intensive glucose-lowering treatment has positive protective significance for patients with a history of CAD. In the available RCTs, for the patients with a history of prior CAD, the total OR value is 0.94 (CI 0.89, 0.99; *P* = 0.002), this treatment had a greater impact than for patients without a history of CAD. The degree of variation can be considered low (*I*^2^ = 0%, *P* = 0.54).

### 3.5. Effects of Intensive Glucose Control and Conventional Glucose Control on Adverse Events at Different Glucose Levels

#### 3.5.1. Considering All the Available RCTs

**Figure 6:**
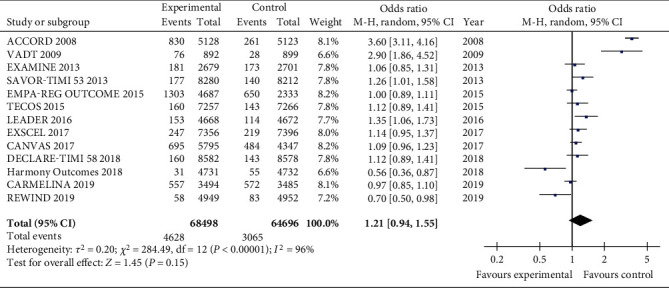
Forest plots of the meta-analyses of the effect of intensive glucose-lowering treatment on adverse events incidence considering all available data.

There was no difference between the two groups when considering all the available RCTs on severe hypoglycemia (Figure 6). When HbA1c decreased by more than 0.5%, the total OR value was 0.98 (CI 0.98, 1.02; *P* = 0.26). The total OR value is 1.21 (CI 0.94, 155; *P* = 0.15) when considering all the studies. Heterogeneity was not significant when all studies were included (*I*^2^ = 96%, *P* < 0.00001).

#### 3.5.2. The Effects of Different Hypoglycemic Degrees on Hypoglycemic Events

**Figure 7:**
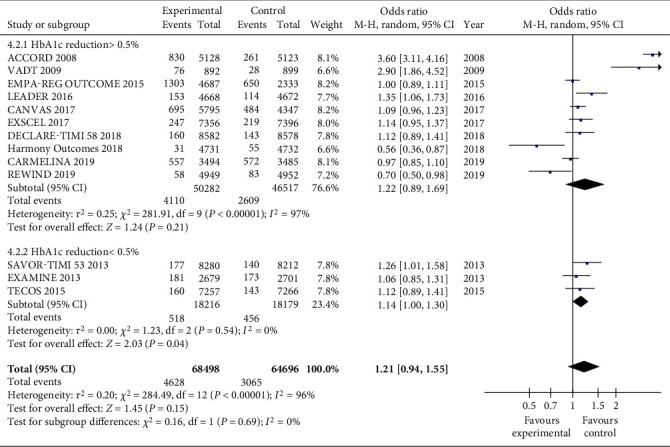
Forest plot for a meta-analysis of the effects of different hypoglycemic degrees on hypoglycemic events.

There was no difference between the two groups when considering the effect of different levels of hypoglycemia on hypoglycemic event (Figure 7). When HbA1c decreased by more than 0.5%, the total OR value was 1.22 (CI 0.89, 1.69; *P* = 0.21). When HbA1c decreased by less than 0.5%, the total OR value was 1.14 (CI 1.00, 1.30; *P* = 0.04). The variation rates of the two subgroups were *I*^2^ = 97% (*P* < 0.00001) and *I*^2^ = 0% (*P* = 0.54), respectively, but there was no difference between the subgroups (*I*^2^ = 0%; *P* = 0.69).

## 4. Discussion

The effect of intensive glycemic control on cardiovascular events is also controversial. Some studies have also shown, such as ACCORD [2], VADT [1], and TECOS [43], that the aim of these studies is to determine the most effective hypoglycemic program, so the mortality rate is higher in the later period. However, whether strict glycemic control has a beneficial effect on the outcome of cardiovascular events, especially myocardial infarction, is critical in determining treatment decisions. At present, many studies are limited to studying the effect of intensive glucose control on MACE, and there are not many studies to analyze the prognostic effect of intensive glucose control on MI. Although the effects of MACE demonstrate clear and long-term benefits of early and intensive glycemic control, and meta-analyses have suggested a “legacy effect” of intensive control [46], data on intensive glycemic control on mi outcomes are conflicting.

The results of this study suggest that, based on existing data, intensive hypoglycemic therapy can effectively reduce the incidence of myocardial infarction in patients with T2DM. In addition, we found that enhanced glucose control had a better protective effect in the group with a reduction of more than 0.5% in HbA1c, but there was no difference between the two groups. At the same time, intensive blood glucose control also has the same effect on MACE, which can effectively reduce the incidence of MACE. In terms of glycemic control, as with MI, a decrease of more than 0.5% in HbA1C compared with a decrease of less than 0.5% in HbA1C did not effectively reduce the incidence of MACE.

The mechanism of hyperglycemia participating in cardiovascular injury is not only the inflammatory injury mentioned above but also related to intestinal microbial thrombosis and sympathetic nerve imbalance. Hyperglycemia in patients with type 2 diabetes, especially under the stressed state of hyperglycemia, is likely to lead to increased thrombotic load in coronary arteries. Earlier, Roberts et al. [47] proposed a new index to evaluate the relative rise of blood sugar. Several studies have demonstrated the predictive value of this indicator in assessing compliance with coronary thrombosis in patients with AMI in both short and long-term outcomes [48]. Studies have shown that glucose is the coordinator of intestinal barrier function, and the hyperglycemia in T2DM patients interferes with the integrity of the homeostasis epithelium, resulting in an abnormal inflow of immunologically stimulating microbial products and a tendency to systemic transmission of intestinal pathogens, resulting in associated inflammation associated with atherosclerosis [49]. And disruption of the balance of the autonomic nervous system may result in catecholamine secretion, calcium transport, and changes in angiotensin II signaling, which may affect cardiac function [50]. As in patients with Takotsubo syndrome (TTS), studies have shown that high blood sugar can have a more negative effect on patients with TTS, possibly because the high blood sugar status impinges on norepinephrine reuptake, leading to more severe and long-lasting myocardial sympathetic denervation [51]. Therefore, the influence of hyperglycemia may also lead to an imbalance between the sympathetic nervous system and the vagus nervous system in patients with T2DM combined with MI, resulting in an increased incidence of MACE events.

The hypoglycemic range has always been controversial, and the fluctuation of HbA1c is generally used to evaluate the hypoglycemic range. Excessive hypoglycemic range can easily lead to hypoglycemic response and increase mortality [52], but a too narrow hypoglycemic range has little effect. In this meta-analysis, subgroup analysis found that for MACE, MI which is the effect was better when HbA1c decreased by more than 0.5%. The possible reason for the above results is that a large decrease in HbA1c can effectively improve the situation of cardiovascular atherosclerosis, resulting in an effective reduction in the incidence of ischemic events. For example, in the trial of VADT (2009) [1], HbA1c decreased from 8.4% to 6.9%. VADT (2015) [28] found that there was a significant decrease in MACE events in the intensive glucose-lowering group compared with the standard treatment group after approximately 11.5 years of follow-up, although the authors thought that there was little effect on the incidence of cardiovascular events and only a benefit for albuminuria. This relative reduction means that 8.6 major cardiovascular events could be prevented per 1000 people/year.

New hypoglycemic drugs that emerged in recent years, such as SGLT2 inhibitors and GLP-1 receptor agonists, have relatively few hypoglycemic events, but in the process of hypoglycemia, there is cardiovascular protection [53]. However, the use of SGLT2 inhibitors had no specific effect on myocardial infarction, stroke, or all-cause mortality and was insignificant compared with the use of GLP-1 agonists. The reason may be that the hypoglycemic effect of SGLT2 inhibitors is not significant, and the main effect may be to improve the ventricular load state, improve cardiac energy metabolism, and inhibit the direct influence of Na+/H+ exchange in the myocardium on the heart [54]. Therefore, SGLT2 inhibitors can effectively reduce the hospitalization rate or mortality rate of heart failure patients but have no significant improvement on atherosclerosis because their hypoglycemic effect is not obvious [55–57]. Long-term use of GLP-1 agonists reduces appetite and anticipated food consumption. Increased satiety and lead to weight loss and reduced plasma LDL cholesterol and triglyceride concentrations, thus reducing the incidence of ischemic events [58, 59]. Moreover, metformin inhibits oxidative low-density lipoprotein absorption and further apoptosis [60], and metformin could activates AMPKa, induces associated telomere amplification, and delays cell senescence, thereby improving endothelial function and further improving the occurrence of cardiovascular events [61]. And alpha-glucosidase inhibitors also improve atherogenic dyslipidemia, reduce insulin resistance, and lead to improved endothelial function, thus reducing cardiovascular events [62].

However, excessive hypoglycemia causing large fluctuations in HbA1c does not reduce the risk but may increase the risk of all-cause mortality. Regardless of baseline HbA1c level, follow-up years, and type of medication, controlling HBA1c to 0.3–0.6% was associated with a significant reduction in hHF, CV, and all-cause mortality, possibly related to the degree of “glycemic change” [63, 64]. In ADVANCE's epidemiological analysis, there was a nonlinear (J-shaped) relationship between mean or baseline HbA1C and mortality in the standard and intensive treatment groups [3]. Another important observational study showed a nonlinear relationship between baseline or mean HbA1C and mortality, involving a large number of middle-aged and elderly patients with type 2 diabetes, in which low HbA1C values were associated with an increased risk of all-cause mortality and cardiovascular events [65]. Although confounding factors such as advanced heart failure or renal failure and malignant tumor were excluded in some patients, the effects of severe hypoglycemia and severe the nutritional damage caused by severe hypoglycemia on CV death, hHF or all-cause death should not be ignored.

### 4.1. Limitations of This Study

We focused on the effect of intensive glucose-lowering treatment on the incidence of myocardial infarction in type 2 diabetes mellitus, but many studies only listed the data on the incidence of myocardial infarction and lacked the data on patients with prior myocardial infarction or acute coronary syndrome after intensive glucose-lowering treatment. Moreover, among patients with cardiovascular disease who are not diagnosed with T2DM, there are also many patients with dysglycaemia. These patients may influence the outcome of intensive hypoglycemic therapy, but data on this are lacking. Many pathological factors, such as inflammation and insulin resistance, have an impact on cardiovascular events, and these factors may influence the results to some extent when blood glucose control is intensified. There was no research on this in the report we included. In addition, as for the fluctuation range of blood glucose, most of the current studies focus on the change of HbA1c, but the change of HbA1c has become more and more controversial in recent years, and more and more clinical attention is paid to glucose variability (GV), which is an important part of blood glucose homeostasis. It can show whether there is excessive blood glucose drift, so as to judge whether there is a risk of hyperglycemia or hypoglycemia. There was no such data in the articles we searched for.

Early multifactorial intensive therapy in patients with T2DM was shown to have a small impact on the incidence of cardiovascular events in a 5-year follow-up trial in ADDITION-Europe in 2011 [66]. A 10-year follow-up study in ADDITION-Europe in 2019 showed no significant difference between multifactor intensive intervention and conservative treatment [67]. A meta-analysis showed that multifactorial interventions had a much greater effect on non-fatal myocardial infarction and non-fatal stroke, but did not reduce or increase the risk of cardiovascular and death outcomes [68]. However, posthoc analysis of the NID-2 trial showed that the number of targeted risk factors was associated with the incidence of cardiovascular events in type 2 diabetes [69]. All the studies included in this study were single-factor intensive therapy, and the included articles did not have relevant data such as blood lipid, blood pressure, and urinary protein after treatment, so the MACE affected by multiple factors could not be studied. This is a limitation of this article.

Finally, the relationship between myocardial infarction and intensive hypoglycemic treatment still needs further study, and the optimal level of HbA1c control in patients after myocardial infarction still needs further discussion.

## 5. Conclusion

Our data support the positive protective effect of glucose-lowering therapy on MI in patients with T2DM, but there is no significant effect of intensive glucose-lowering. In terms of MACE, hypoglycemic treatment also had a similar protective effect on patients with T2DM, but intensive hypoglycemic treatment had no significant effect. In addition, we found no greater protective effect of enhanced glucose control in the HbA1c reduction of more than 0.5%, and no difference in the incidence of adverse events compared with the HbA1c reduction of less than 0.5%.

## Supplementary Materials

Supplementary Materials Supplementary Figure 1: flow diagram of literature selection. Supplementary Figure 2: risk of bias graph for the judgement about each methodological quality item that presented as percentages across all included studies. Supplementary Figure 3: risk of bias summary for all of the judgements about risk of bias for all included studies. Supplementary Figure 4: funnel plots and Egger's regression asymmetry test for assessing publication bias (a-MI, b-MACE, c-All-cause death, d-Severe hypoglycaemia). Table 1:the main features of the observational studies. Table 2: search strategy of relevant literature. Table 3: the history of past cardiovascular disease of the observational studies. Table 4: the oral anticardiovascular drug use before admission.
