# Supplementary material for: Effect of Intensive Glycemic Control on Myocardial Infarction Outcome in Patients with Type 2 Diabetes Mellitus: A Systematic Review and Meta-Analysis
Source: J Diabetes Res. 2023 Feb 24;2023:8818502. doi: 10.1155/2023/8818502 (PMC9984264; doi:10.1155/2023/8818502)
Supplement: Supplementary Materials — Supplementary Figure 1: flow diagram of literature selection. Supplementary Figure 2: risk of bias graph for the judgement about each methodological quality item that presented as percentages across all included studies. Supplementary Figure 3: risk of bias summary for all of the judgements about risk of bias for all included studies. Supplementary Figure 4: funnel plots and Egger's regression asymmetry test for assessing publication bias (a-MI, b-MACE, c-All-cause death, d-Severe hypoglycaemia). Table 1:the main features of the observational studies. Table 2: search strategy of relevant literature. Table 3: the history of past cardiovascular disease of the observational studies. Table 4: the oral anticardiovascular drug use before admission. [file 8818502.f1.zip › Supplementary Figure 3 (1).pdf]

	Random sequence generation (selection bias)	Allocation concealment (selection bias)	Blinding of participants and personnel (performance bias)	Blinding of outcome assessment (detection bias)	Incomplete outcome data (attrition bias)	Selective reporting (reporting bias)	Other bias
ACCORD 2008	+	+	+	?	+	?	+
ADVANCE 2008	+	+	+	?	+	?	+
CANVAS 2017	+	+	?	?	+	?	+
CARMELINA 2019	+	+	+	+	+	+	+
DECLARE – TIMI 58 2018	+	+	+	+	+	+	+
EMPA-REG OUTCOME 2015	+	+	+	+	+	+	+
EXAMINE 2013	?	?	+	+	+	+	+
EXSCEL 2017	+	?	+	+	+	?	?
Harmony Outcomes 2018	+	+	+	?	+	?	?
LEADER 2016	?	?	+	+	+	+	?
REWIND 2019	+	+	+	+	+	+	+
SAVOR-TIMI 53 2013	+	+	+	+	+	+	+
TECOS 2015	?	+	+	+	+	+	+
VADT 2009	+	-	?	?	+	?	?
