# Supplementary material for: Effect of Intensive Glycemic Control on Myocardial Infarction Outcome in Patients with Type 2 Diabetes Mellitus: A Systematic Review and Meta-Analysis
Source: J Diabetes Res. 2023 Feb 24;2023:8818502. doi: 10.1155/2023/8818502 (PMC9984264; doi:10.1155/2023/8818502)
Supplement: Supplementary Materials — Supplementary Figure 1: flow diagram of literature selection. Supplementary Figure 2: risk of bias graph for the judgement about each methodological quality item that presented as percentages across all included studies. Supplementary Figure 3: risk of bias summary for all of the judgements about risk of bias for all included studies. Supplementary Figure 4: funnel plots and Egger's regression asymmetry test for assessing publication bias (a-MI, b-MACE, c-All-cause death, d-Severe hypoglycaemia). Table 1:the main features of the observational studies. Table 2: search strategy of relevant literature. Table 3: the history of past cardiovascular disease of the observational studies. Table 4: the oral anticardiovascular drug use before admission. [file 8818502.f1.zip › Supplementary Figure.docx]

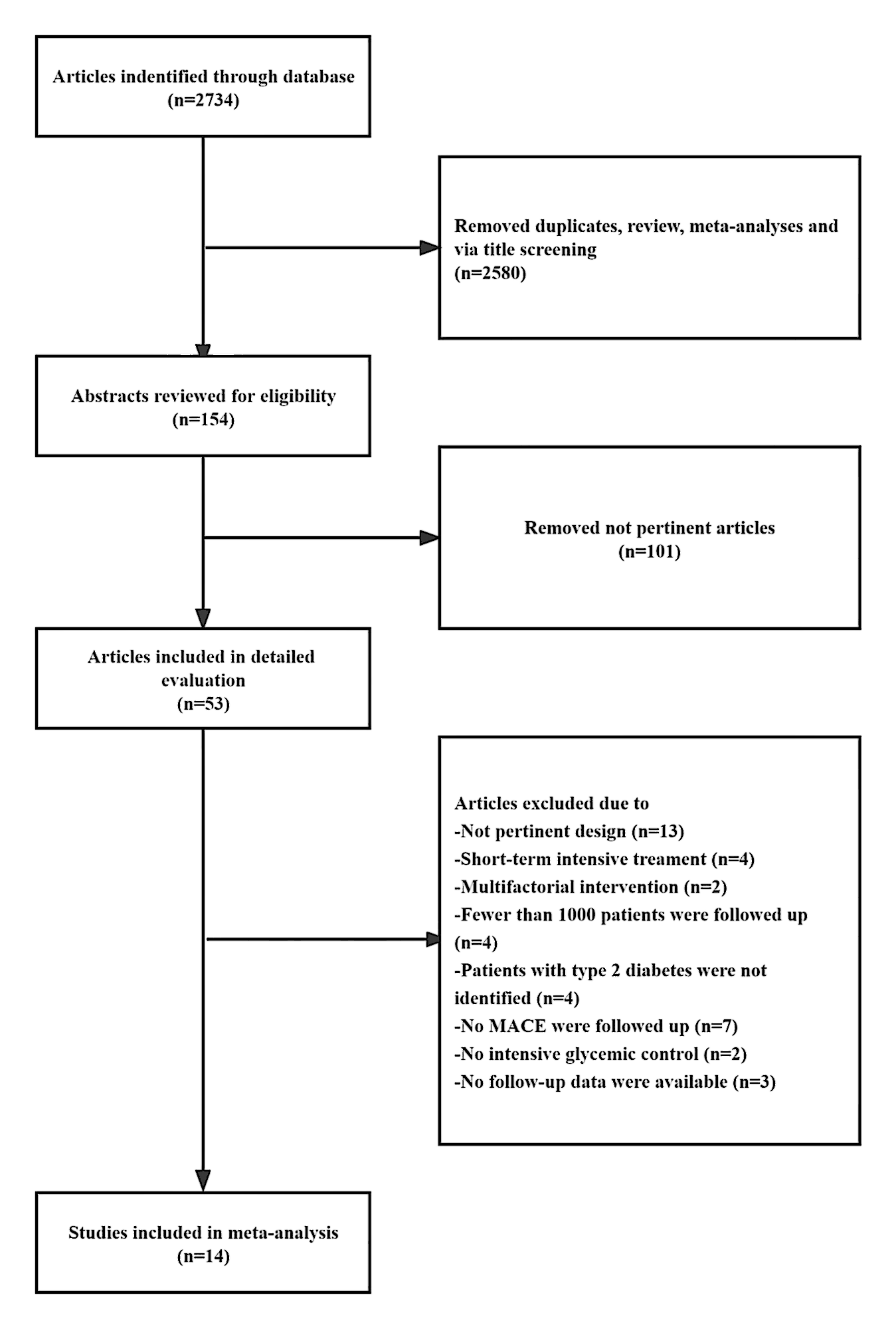


**Supplementary Figure 1. Flow diagram of literature selection**


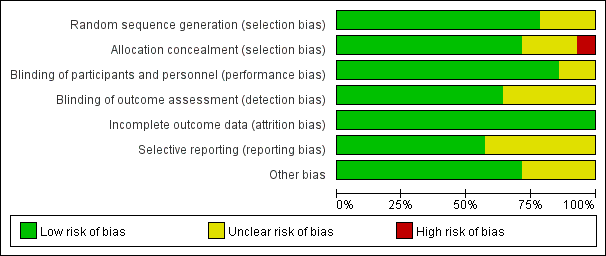


**Supplementary Figure 2. Risk of bias graph for the judgement about each methodological quality item that presented as percentages across all included studies**

**
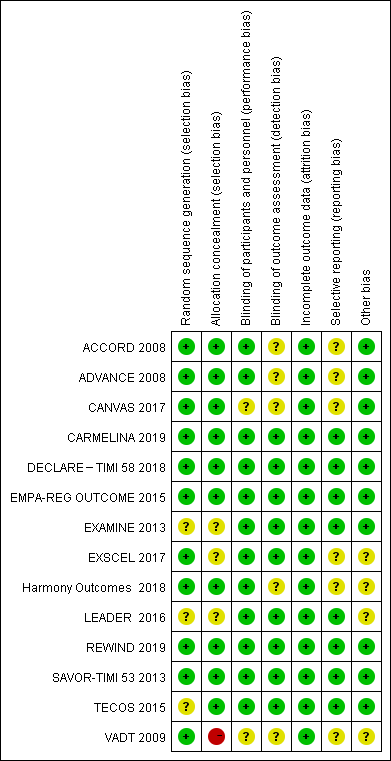
**

**Supplementary Figure 3. Risk of bias summary for all of the judgements about risk of bias for all included studies**


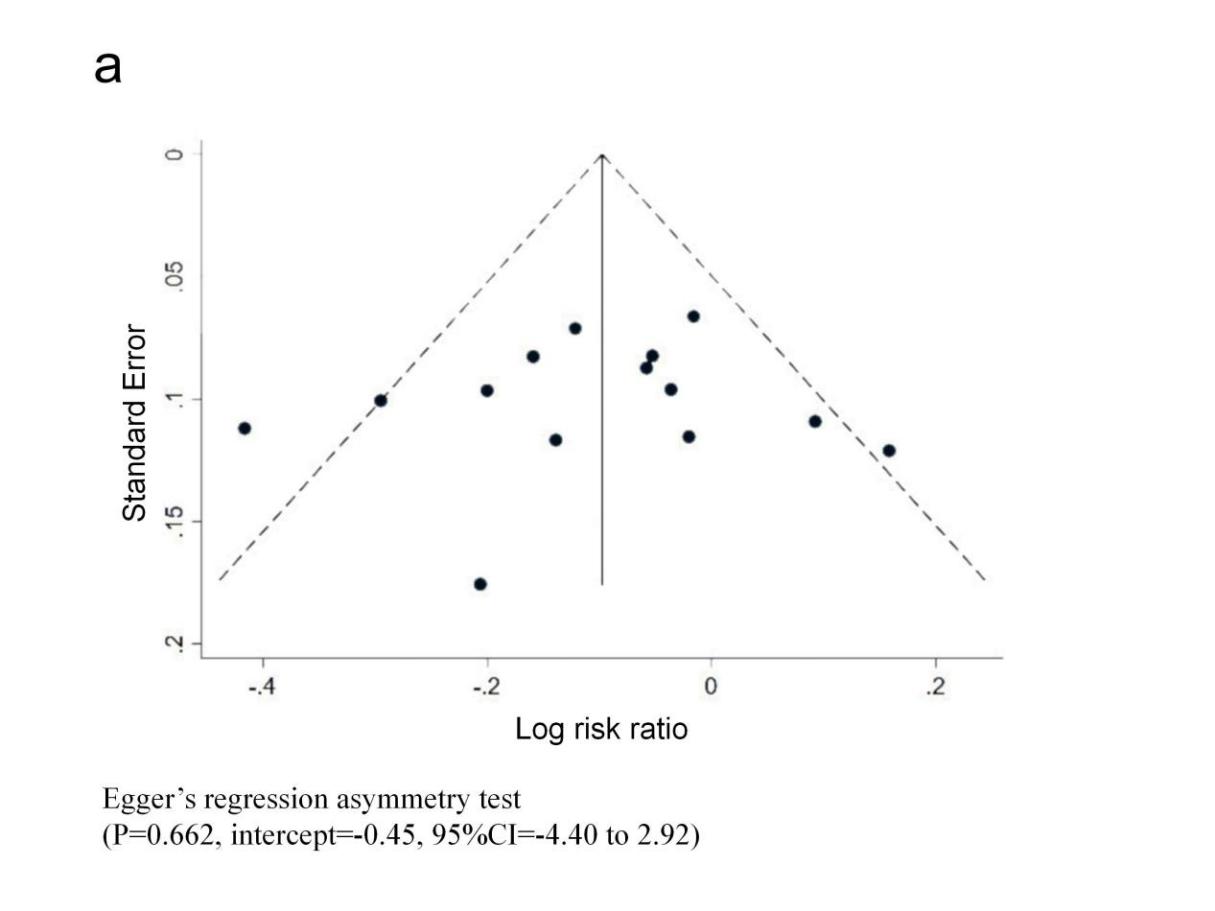


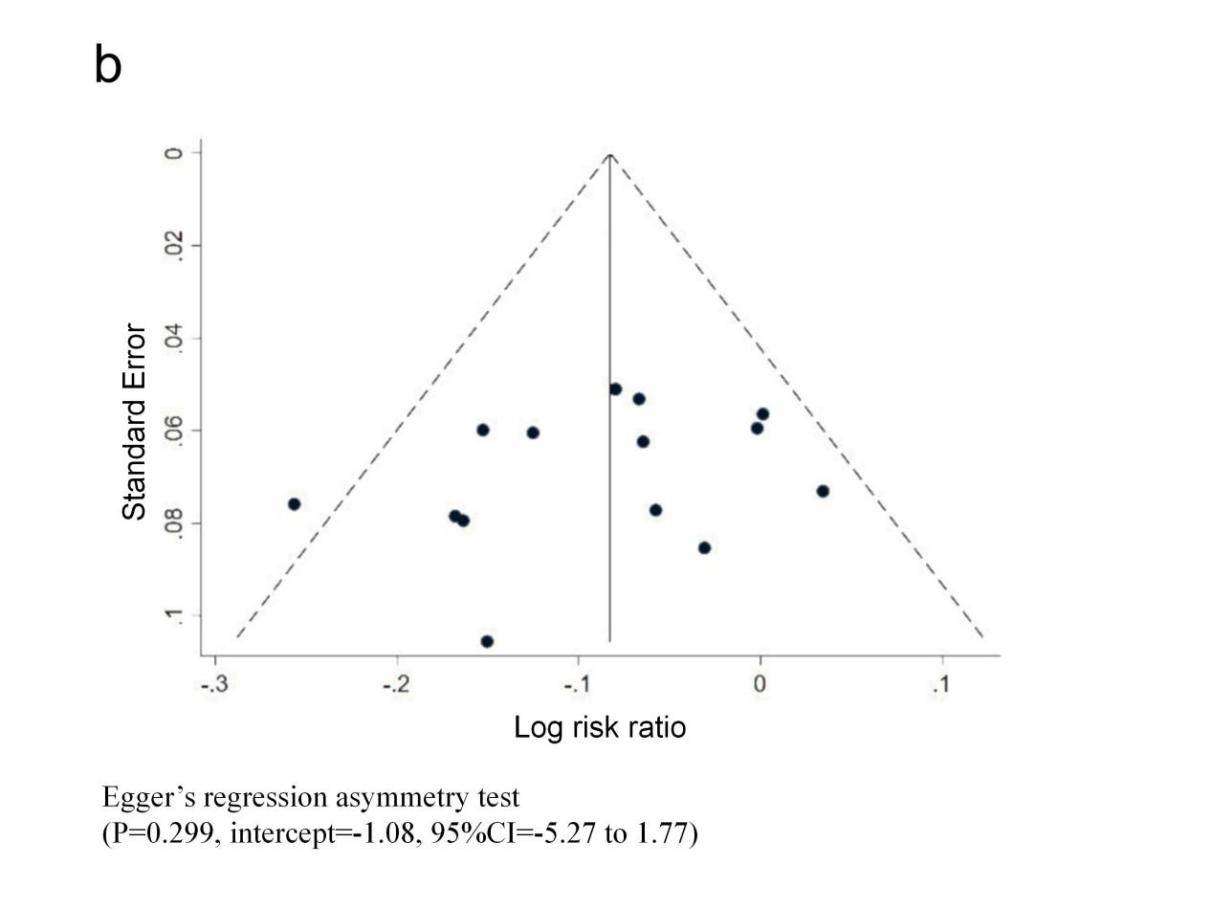


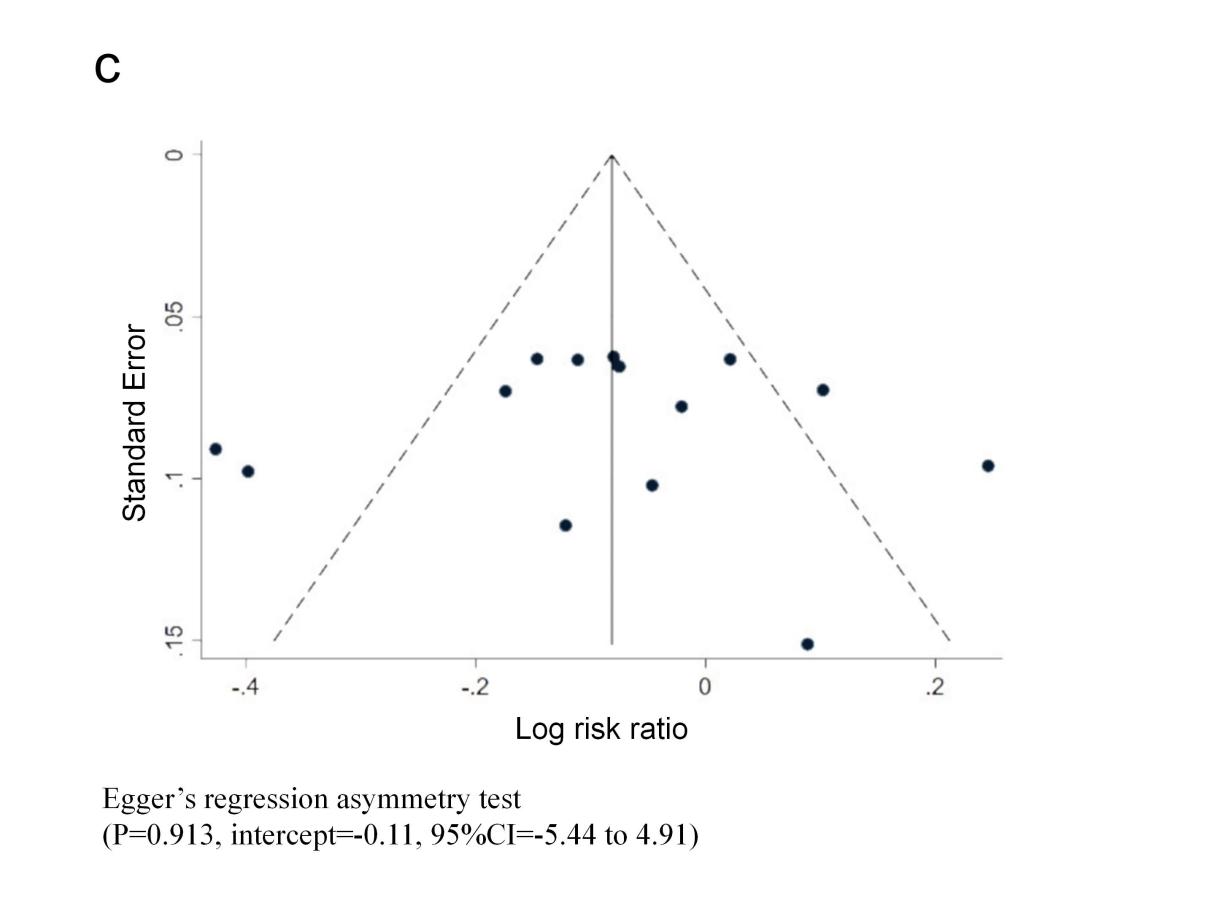


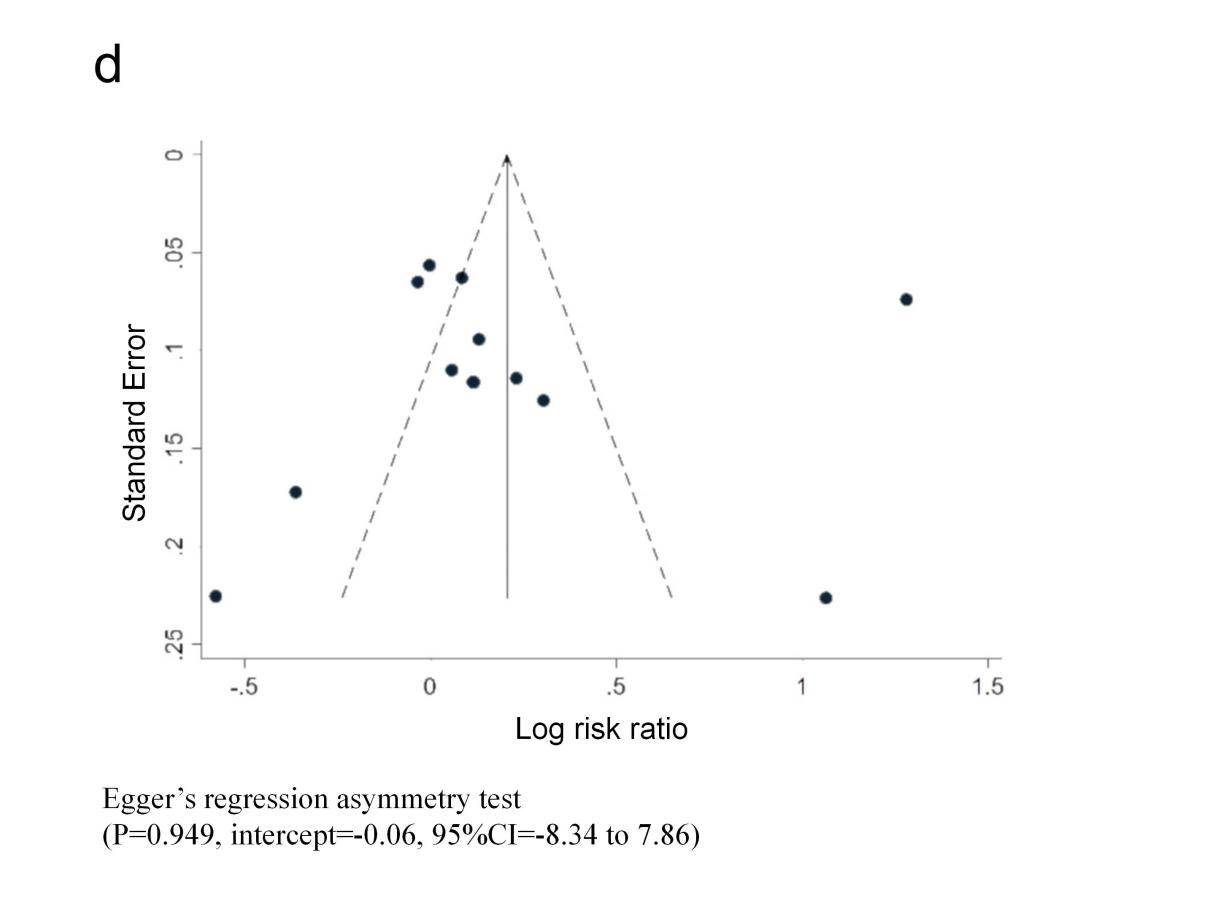


**Supplementary Figure 4. Funnel plots and Egger’s regression asymmetry test for assessing publication bias(a-MI, b-MACE, c-All-cause death, d-Severe hypoglycaemia)**
