# Supplementary figures and images for: Effect of Intensive Glycemic Control on Myocardial Infarction Outcome in Patients with Type 2 Diabetes Mellitus: A Systematic Review and Meta-Analysis
Source: J Diabetes Res. 2023 Feb 24;2023:8818502. doi: 10.1155/2023/8818502 (PMC9984264; doi:10.1155/2023/8818502)

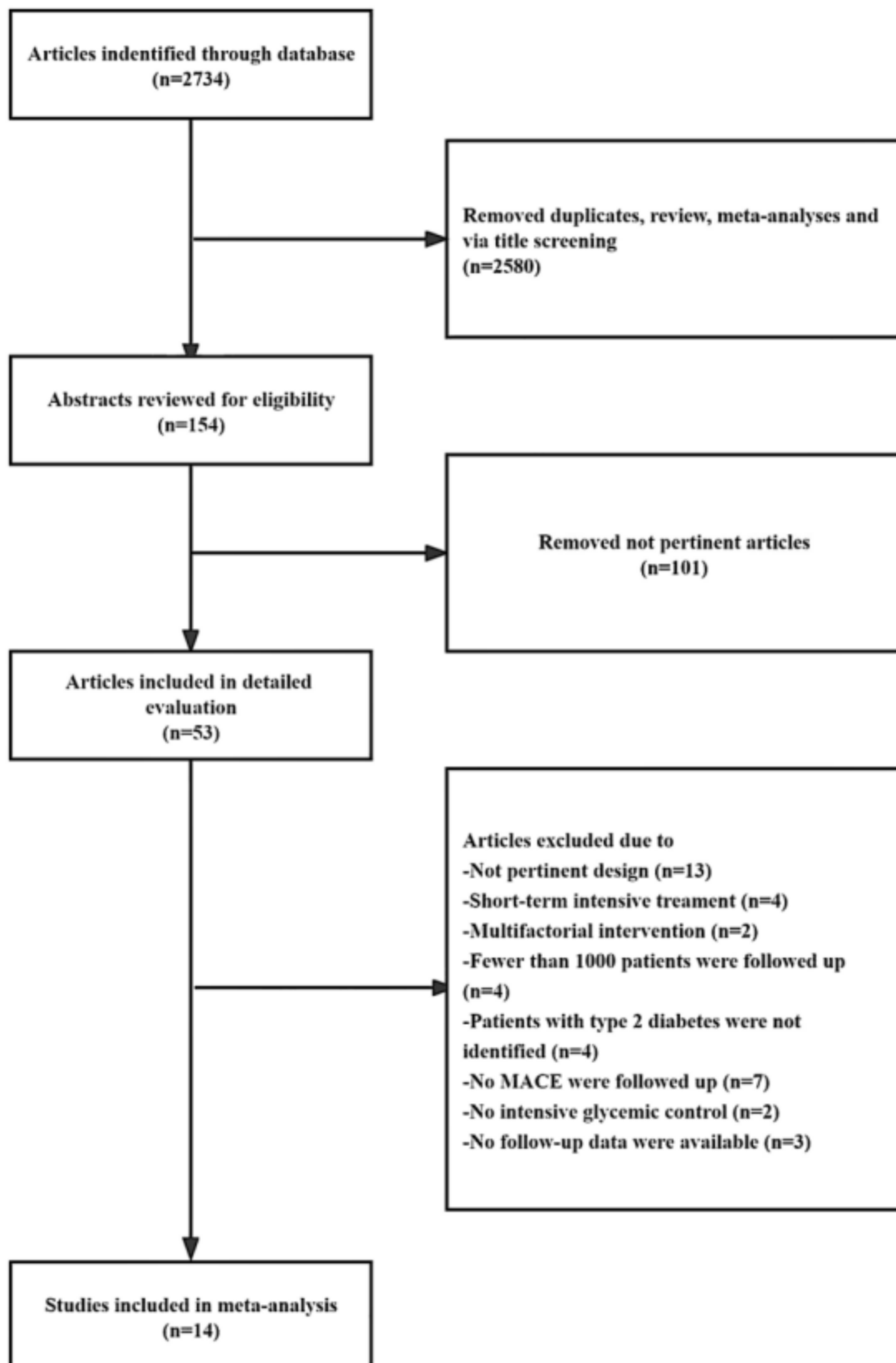

Supplement: Supplementary Materials — Supplementary Figure 1: flow diagram of literature selection. Supplementary Figure 2: risk of bias graph for the judgement about each methodological quality item that presented as percentages across all included studies. Supplementary Figure 3: risk of bias summary for all of the judgements about risk of bias for all included studies. Supplementary Figure 4: funnel plots and Egger's regression asymmetry test for assessing publication bias (a-MI, b-MACE, c-All-cause death, d-Severe hypoglycaemia). Table 1:the main features of the observational studies. Table 2: search strategy of relevant literature. Table 3: the history of past cardiovascular disease of the observational studies. Table 4: the oral anticardiovascular drug use before admission. [file 8818502.f1.zip › Supplementary Figure 1 (1).pdf]

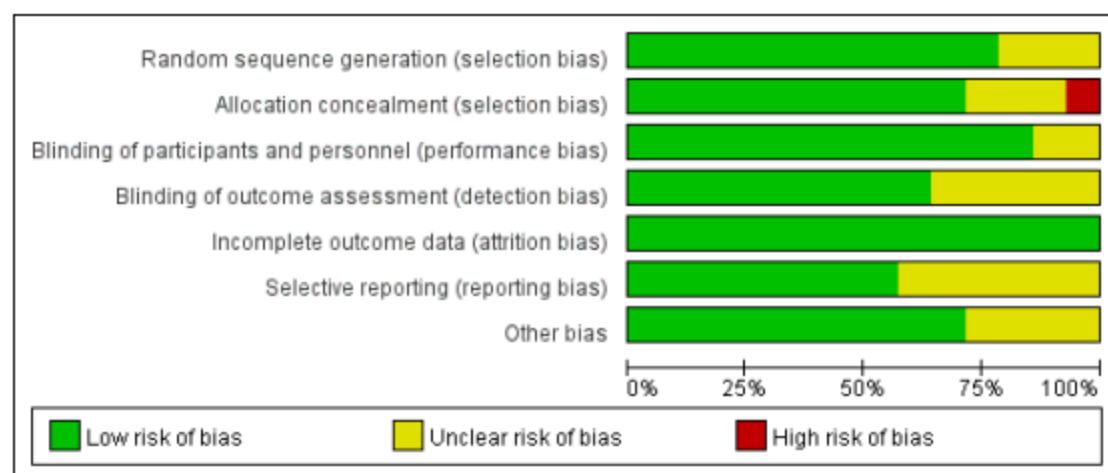

Supplement: Supplementary Materials — Supplementary Figure 1: flow diagram of literature selection. Supplementary Figure 2: risk of bias graph for the judgement about each methodological quality item that presented as percentages across all included studies. Supplementary Figure 3: risk of bias summary for all of the judgements about risk of bias for all included studies. Supplementary Figure 4: funnel plots and Egger's regression asymmetry test for assessing publication bias (a-MI, b-MACE, c-All-cause death, d-Severe hypoglycaemia). Table 1:the main features of the observational studies. Table 2: search strategy of relevant literature. Table 3: the history of past cardiovascular disease of the observational studies. Table 4: the oral anticardiovascular drug use before admission. [file 8818502.f1.zip › Supplementary Figure 2 (1).pdf]

a

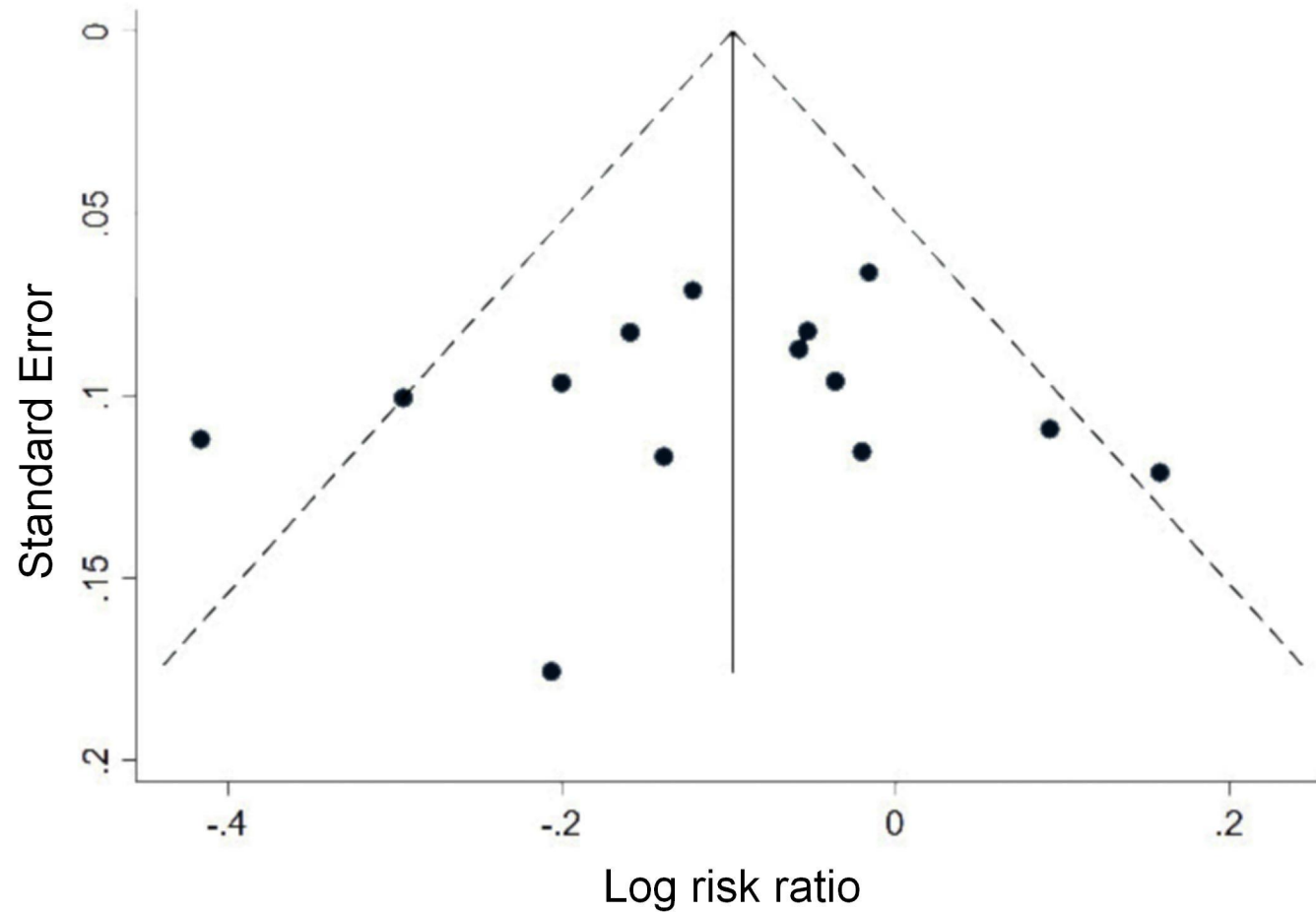

Egger's regression asymmetry test  
( $P=0.662$ , intercept=-0.45, 95%CI=-4.40 to 2.92)

Supplement: Supplementary Materials — Supplementary Figure 1: flow diagram of literature selection. Supplementary Figure 2: risk of bias graph for the judgement about each methodological quality item that presented as percentages across all included studies. Supplementary Figure 3: risk of bias summary for all of the judgements about risk of bias for all included studies. Supplementary Figure 4: funnel plots and Egger's regression asymmetry test for assessing publication bias (a-MI, b-MACE, c-All-cause death, d-Severe hypoglycaemia). Table 1:the main features of the observational studies. Table 2: search strategy of relevant literature. Table 3: the history of past cardiovascular disease of the observational studies. Table 4: the oral anticardiovascular drug use before admission. [file 8818502.f1.zip › Supplementary Figure 4(a) (1).pdf]

b

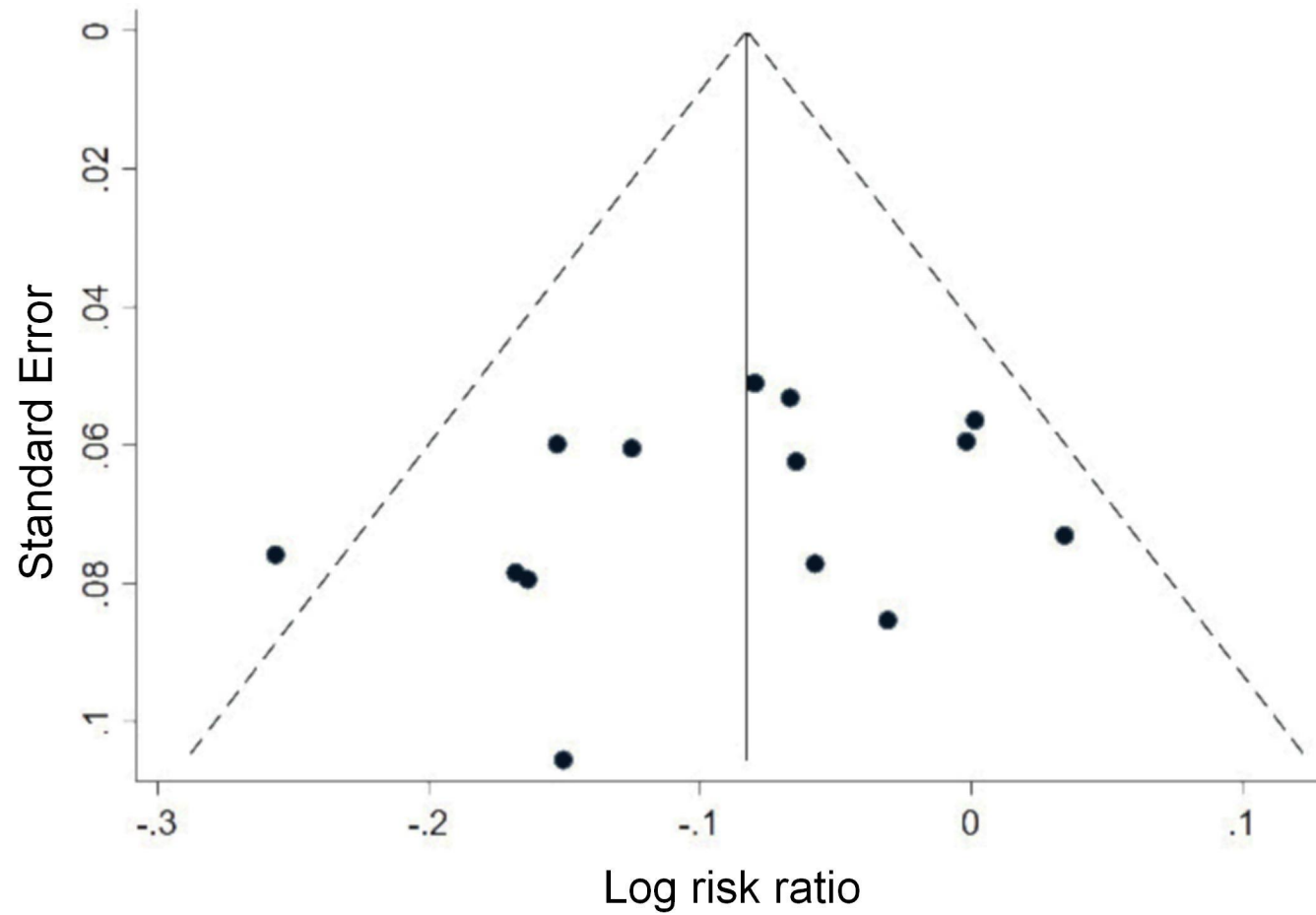

Egger's regression asymmetry test  
( $P=0.299$ , intercept=-1.08, 95%CI=-5.27 to 1.77)

Supplement: Supplementary Materials — Supplementary Figure 1: flow diagram of literature selection. Supplementary Figure 2: risk of bias graph for the judgement about each methodological quality item that presented as percentages across all included studies. Supplementary Figure 3: risk of bias summary for all of the judgements about risk of bias for all included studies. Supplementary Figure 4: funnel plots and Egger's regression asymmetry test for assessing publication bias (a-MI, b-MACE, c-All-cause death, d-Severe hypoglycaemia). Table 1:the main features of the observational studies. Table 2: search strategy of relevant literature. Table 3: the history of past cardiovascular disease of the observational studies. Table 4: the oral anticardiovascular drug use before admission. [file 8818502.f1.zip › Supplementary Figure 4(b) (1).pdf]

C

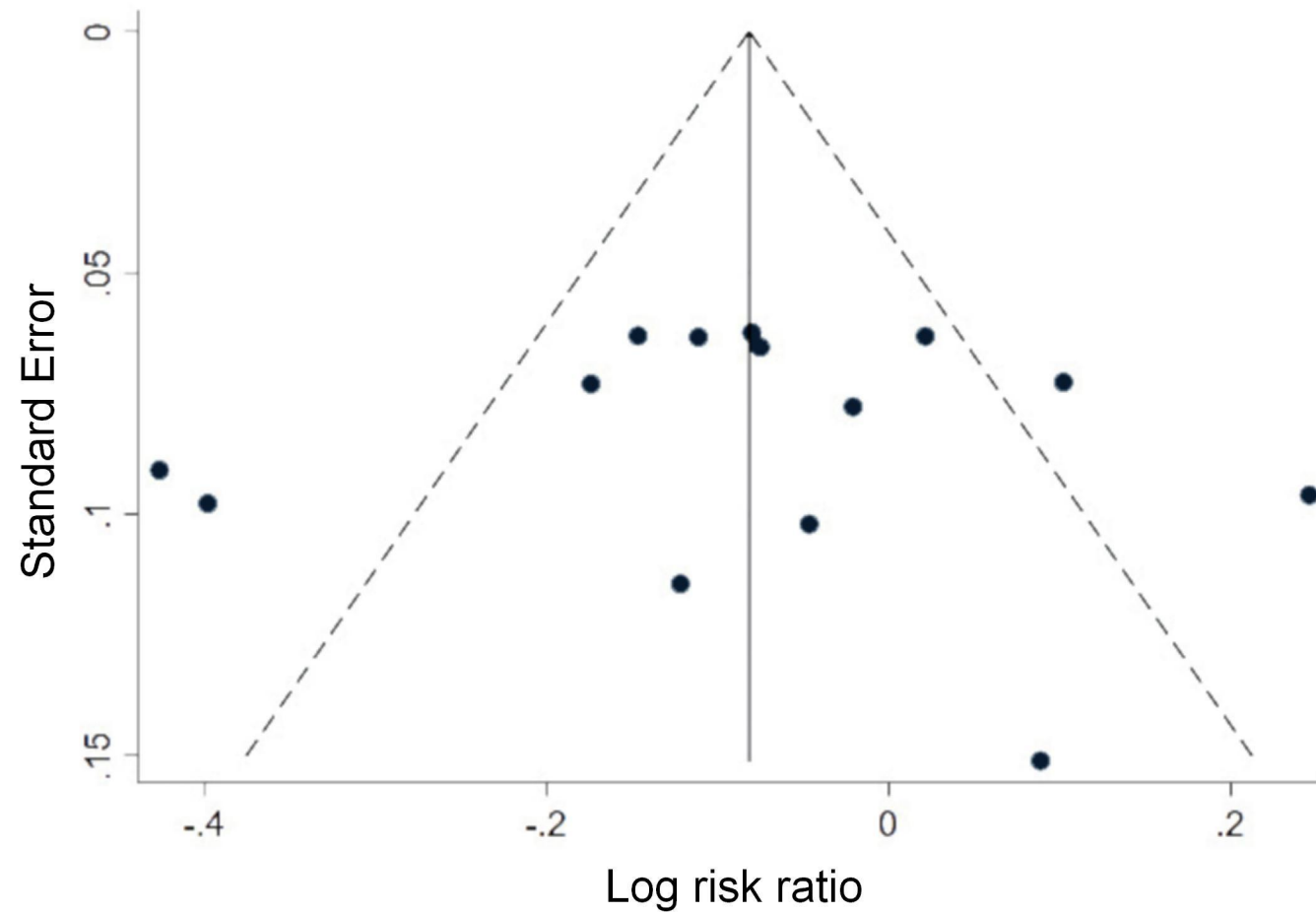

Egger's regression asymmetry test  
( $P=0.913$ , intercept=-0.11, 95%CI=-5.44 to 4.91)

Supplement: Supplementary Materials — Supplementary Figure 1: flow diagram of literature selection. Supplementary Figure 2: risk of bias graph for the judgement about each methodological quality item that presented as percentages across all included studies. Supplementary Figure 3: risk of bias summary for all of the judgements about risk of bias for all included studies. Supplementary Figure 4: funnel plots and Egger's regression asymmetry test for assessing publication bias (a-MI, b-MACE, c-All-cause death, d-Severe hypoglycaemia). Table 1:the main features of the observational studies. Table 2: search strategy of relevant literature. Table 3: the history of past cardiovascular disease of the observational studies. Table 4: the oral anticardiovascular drug use before admission. [file 8818502.f1.zip › Supplementary Figure 4(c) (1).pdf]

a

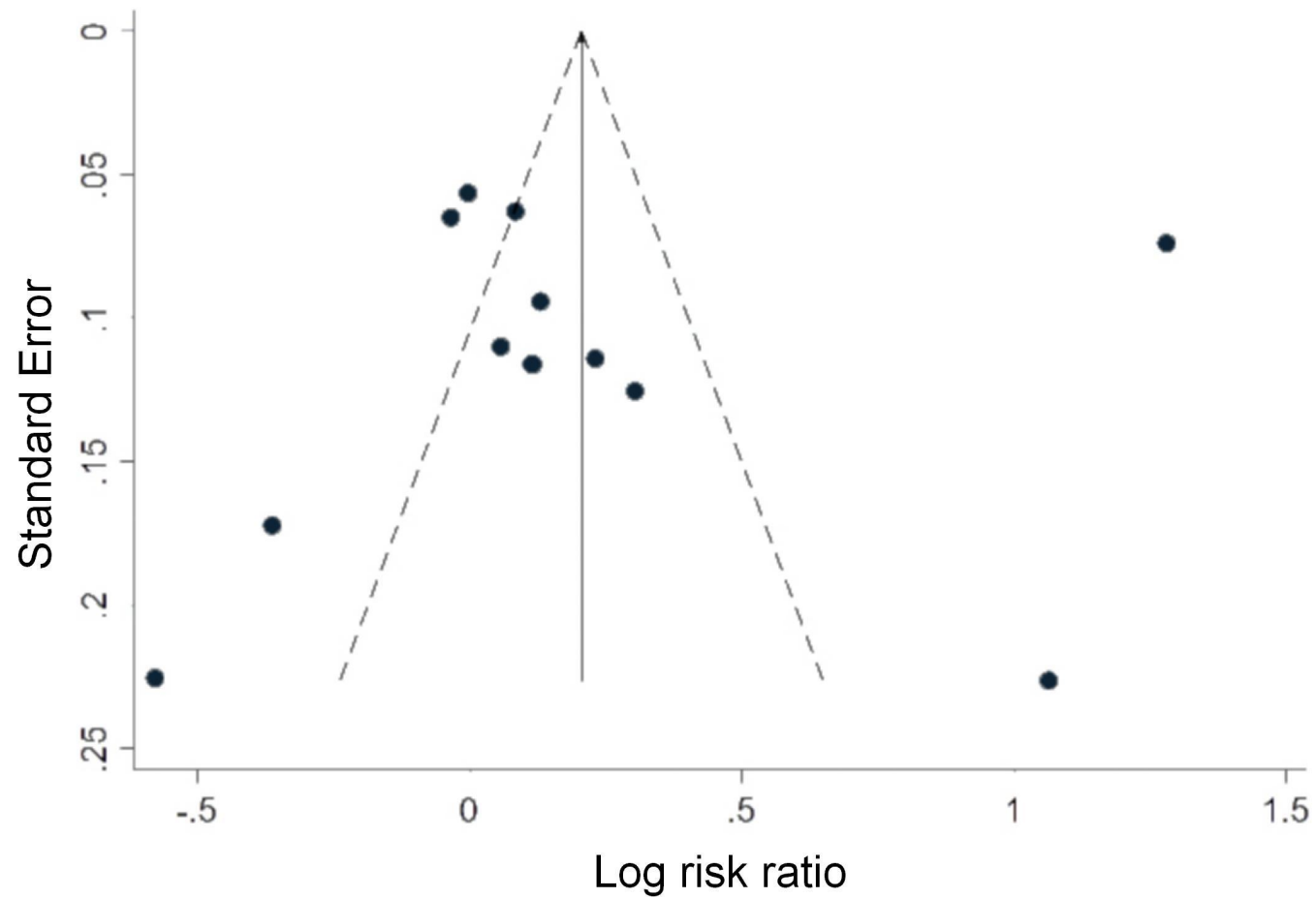

Egger's regression asymmetry test  
( $P=0.949$ , intercept=-0.06, 95%CI=-8.34 to 7.86)

Supplement: Supplementary Materials — Supplementary Figure 1: flow diagram of literature selection. Supplementary Figure 2: risk of bias graph for the judgement about each methodological quality item that presented as percentages across all included studies. Supplementary Figure 3: risk of bias summary for all of the judgements about risk of bias for all included studies. Supplementary Figure 4: funnel plots and Egger's regression asymmetry test for assessing publication bias (a-MI, b-MACE, c-All-cause death, d-Severe hypoglycaemia). Table 1:the main features of the observational studies. Table 2: search strategy of relevant literature. Table 3: the history of past cardiovascular disease of the observational studies. Table 4: the oral anticardiovascular drug use before admission. [file 8818502.f1.zip › Supplementary Figure 4(d) (1).pdf]
