# Supplementary material for: Effect of Intensive Glycemic Control on Myocardial Infarction Outcome in Patients with Type 2 Diabetes Mellitus: A Systematic Review and Meta-Analysis
Source: J Diabetes Res. 2023 Feb 24;2023:8818502. doi: 10.1155/2023/8818502 (PMC9984264; doi:10.1155/2023/8818502)
Supplement: Supplementary Materials — Supplementary Figure 1: flow diagram of literature selection. Supplementary Figure 2: risk of bias graph for the judgement about each methodological quality item that presented as percentages across all included studies. Supplementary Figure 3: risk of bias summary for all of the judgements about risk of bias for all included studies. Supplementary Figure 4: funnel plots and Egger's regression asymmetry test for assessing publication bias (a-MI, b-MACE, c-All-cause death, d-Severe hypoglycaemia). Table 1:the main features of the observational studies. Table 2: search strategy of relevant literature. Table 3: the history of past cardiovascular disease of the observational studies. Table 4: the oral anticardiovascular drug use before admission. [file 8818502.f1.zip › Supplementary Table (1).doc]

**Supplemental Table**

**Table 1.** The main features of the observational studies

**Table 2.** Search strategy of relevant literature

**Table 3**. The history of past cardiovascular disease of the observational studies

**Table 4**. The oral anti-cardiovascular drug use before admission

| Trial | Years | Participants  (Int/Con) | Drugs | Mean follow-up(y) | Mean age  (y) | Duration of diabetes  (y) | HbA1c, %  (Baseline) | Achieved,%  (Int/Cont) | Mean reduction in achieved level |
| --- | --- | --- | --- | --- | --- | --- | --- | --- | --- |
| REWIND | 2019 | 4949/4952 | Dulaglutide | 5.4 | 66.2 | 10.5 | 7.20 | 6.60/7.21 | -0.61% |
| DECLARE–TIMI 58 | 2019 | 8582/8578 | Dapagliflozin | 4.2 | 64 | 11 | 8.30 | 7.90/8.10 | -0.20% |
| CARMELINA | 2019 | 34943485 | Linagliptin | 2.2 | 65.85 | 14.75 | 7.9/8.0 | 7.38/7.89 | -0.51% |
| Harmony Outcomes | 2018 | 4731/4732 | Albiglutide | 1.6 | 64.15 | 14.15 | 8.74 | 7.70/8.50 | -0.90% |
| CANVAS | 2017 | 5795/4347 | Canagliflozin | 2.4 | 63.3 | 13.6 | 8.20 | 7.52/8.15 | -0.63% |
| EXSCEL | 2017 | 7356/7396 | Exenatide | 3.2 | 62 | 12.0 | 8.00 | 7.30/8.00 | -0.70% |
| LEADER | 2016 | 4668/4672 | Liraglutide | 3.8 | 64.3 | 12.85 | 8.70 | 7.20/8.20 | -1.00% |
| EMPA-REG OUTCOME | 2015 | 4687/2333 | Empagliflozin | 3.1 | 63.15 | - | 8.08 | 7.45/8.00 | -0.54% |
| TECOS | 2015 | 7332/7339 | Sitaglipti | 3.0 | 65.5 | 11.6 | 7.20 | 6.90/7.30 | -0.40% |
| SAVOR-TIMI 53 | 2013 | 8280/8212 | Saxagliptin | 2.1 | 65.05 | 10.3 | 8.00 | 7.50/7.80 | -0.30% |
| EXAMINE | 2013 | 2701/2679 | Alogliptin | 1.5 | 61 | 7.2 | 8.00 | 7.67/7.97 | -0.30% |
| VADT | 2009 | 892/899 | comprehensive | 5.6 | 60.4 | 11.5 | 9.40 | 6.90/8.40 | -1.50% |
| ADVANCE | 2008 | 5571/5569 | comprehensive | 5.0 | 66.0 | 7.95 | 7.51/7.52 | 6.50/7.30 | -0.79% |
| ACCORD | 2008 | 5128/5123 | comprehensive | 3.5 | 62.2 | 10 | 8.30 | 6.70/7.50 | -0.80% |

**Supplementary Table 1. The main features of the observational studies**

**Supplementary Table 2. Search strategy of relevant literature**

1. MEDLINE, PreMEDLINE, and other related databases via PubMed search interface

| #1 | Search "Diabetes Mellitus, Type 2"[Mesh] |
| --- | --- |
| #2 | Search "Type 2 Diabetes" OR "Non-Insulin-Dependent Diabetes Mellitus" OR (type 2 diabetic*) OR “Stable Diabetes Mellitus” |
| #3 | Search (#1 OR #2) |
| #4 | Search "Glycemic Control"[Mesh] |
| #5 | Search (intensive glucose control*) OR (blood glucose target*) OR (Blood Glucose Control*) |
| #6 | Search (#4 OR #5) |
| #7 | Search ("Cardiovascular Diseases"[Mesh]) OR "Myocardial Infarction"[Mesh]) OR "Stroke"[Mesh]) OR "Brain Ischemia"[Mesh]) OR "Intracranial Embolism and Thrombosis"[Mesh]) OR "Intracranial Hemorrhages"[Mesh]) OR "Heart Failure"[Mesh] |
| #8 | Search (major adverse cardiovascular event) OR "Coronary heart disease" OR "myocardial infarction" OR "heart failure" OR "cardiovascular death" OR "cardiovascular deaths" OR "cardiovascular mortality" OR "all-cause death" OR "all-cause deaths" OR "all-cause mortality" |
| #9 | Search (#7 OR #8) |
| #10 | Search (#3 AND #6 AND #9) |
| #11 | Search (#3 AND #6 AND #9) Filters: Randomized Controlled Trial; Controlled Clinical Trial; Clinical Trial |

2. Cochrane Library via Wiley search interface

| #1 | MeSH descriptor: [Diabetes Mellitus, Type 2] explode all trees |
| --- | --- |
| #2 | "Type 2 Diabetes" OR "Non-Insulin-Dependent Diabetes Mellitus" OR (type 2 diabetic*) OR “Stable Diabetes Mellitus”(Word variations have been searched) |
| #3 | #1 or #2 |
| #4 | MeSH descriptor: [Glycemic Control] explode all trees |
| #5 | Search (intensive glucose control*) OR (blood glucose target*) OR (Blood Glucose Control*) (Word variations have been searched) |
| #6 | #4 or #5 |
| #7 | MeSH descriptor: [Cardiovascular Diseases] explode all trees |
| #8 | (major adverse cardiovascular event) OR "Coronary heart disease" OR "myocardial infarction" OR "heart failure" OR "cardiovascular death" OR "cardiovascular deaths" OR "cardiovascular mortality" OR "all-cause death" OR "all-cause deaths" OR "all-cause mortality" (Word variations have been searched) |
| #9 | #7 or #8 |
| #10 | #3 and #6 and #9 in Trials |

| Trial | Years | Participants  (Int/Con) | [previous history](javascript:;)  (Int/Con) |
| --- | --- | --- | --- |
| REWIND | 2019 | 4949/4952 | Cardiovascular disease 1560/1554  Cardiovascular event 1028/1007 |
| DECLARE–TIMI 58 | 2019 | 8582/8578 | History of coronary artery disease 2824/2834  History of cerebrovascular disease 653/648  History of heart failure 852/872 |
| CARMELINA | 2019 | 3494/3485 | History of ischemic heart disease 2029/2052  History of hypertension 3171/3178  Atrial fibrillation 319/354 |
| Harmony Outcomes | 2018 | 4731/4732 | History of Coronary artery disease 3333/3345  History of Hypertension 4089/4095  History of Myocardial infarction 2223/2236  Coronary artery bypass surgery 890/842  Percutaneous coronary intervention 2050/2113  History of Stroke 827/854  History of Heart failure 954/968 Atrial fibrillatio 394/392 |
| CANVAS | 2017 | 5795/4347 | History of hypertension 5188/3937  History of heart failure 803/858  History of Coronary vascular disease 3234/2487  History of cardiovascular disease 3756/2900 |
| EXSCEL | 2017 | 7356/7396 | History of Coronary artery disease 3898/3896  History of congestive heart failure 1161/1228  Prior CV event at randomization 5394/5388 |
| LEADER | 2016 | 4668/4672 | Prior myocardial infarction 1464/1400  Prior stroke or transient ischemic attack 730/777  >50% stenosis of coronary 1188/1191  Heart failure NYHA II – III 653/652 |
| EMPA-REG OUTCOME | 2015 | 4687/2333 | History of Coronary artery disease 3545/1763  Multi-vessel coronary artery disease 2179/1100  History of myocardial infarction 2190/1083  Coronary artery bypass graft 1175/563  History of stroke 1084/553  Cardiac failure 462/244 |
| TECOS | 2015 | 7332/7339 | Prior cardiovascular disease 5397/5466  Myocardial infarction 3133/3122  >50% coronary stenosis 3804/3883  Prior PCI 2814/2900  CABG 1845/1819  Prior cerebrovascular disease 1806/1782  Prior Congestive heart failure 1303/1340 |
| SAVOR-TIMI 53 | 2013 | 8280/8212 | Prior myocardial infarction 3147/3090  Prior heart failure 1056/1049  Prior coronary revascularization 3566/3557 |
| EXAMINE | 2013 | 2701/2679 | History of Myocardial infarction 2389/2345  Percutaneous coronary intervention 1689/1683  Coronary-artery bypass grafting 347/341  Hypertension 2229/2240  Congestive heart failure 757/744  Stroke 195/193 |
| VADT | 2009 | 892/899 | Patients with previous cardiovascular event 355/368  Patients with hypertension 642/650 |
| ADVANCE | 2008 | 5571/5569 | History of major macrovascular disease 1794/1796  Myocardial infarction 668/666  Stroke 515/508 |
| ACCORD | 2008 | 5128/5123 | Previous cardiovascular event 1826/1783  Previous congestive heart failure 251/246 |

**Supplementary Table 3. The history of past cardiovascular disease of the observational studies**

| Trial | Years | Participants  (Int/Con) | ACE inhibitor or ARB | β blocker | Diuretics | Calcium antagonists | Antiplatelet | Statin or ezetimibe |
| --- | --- | --- | --- | --- | --- | --- | --- | --- |
| REWIND | 2019 | 4949/4952 | 4009/4059 | 2237/2274 | — | — | 2662/2680 | — |
| DECLARE–TIMI 58 | 2019 | 8582/8578 | 6977/6973 | 4498/4532 | 3488/3479 | — | 5245/5242 | 6432/6446 |
| CARMELINA | 2019 | 3494/3485 | 2860/2798 | 2080/2073 | 1892/1936 | 1433/1446 | 2166/2178  (Aspirin) | 2495/2523 |
| Harmony Outcomes | 2018 | 4731/4732 | ACEI:2263/2353  ARB:1599/1511 | 3128/3182 | 1428/1431 | 1428/1431 | 3652/3639  (Aspirin) | 3967/3988 |
| CANVAS | 2017 | 5795/4347 | 4645/3471 | 3039/2382 | 2536/1954 | — | 4233/3233 | 4329/3270 |
| EXSCEL | 2017 | 7356/7396 | ACEI:3535/3647  ARB:2334/2272 | 4082/4129 | 3216/3227 | 2380/2330 | Aspirin:4713/4667  Clopidogrel:1255/1269 | Statin:5465/5380  Ezetimibe:347/364 |
| LEADER | 2016 | 4668/4672 | ACEI:2417/2350  ARB:1488/1486 | 2652/2529 | 1953/1953 | 1538/1479 | Aspirin:2977/2899  Clopidogrel:720/745 | 3405/3336 |
| EMPA-REG OUTCOME | 2015 | 4687/2333 | 3798/1868 | 3056/1498 | 2047/988 | 1529/788 | Aspirin:3876/1927  Clopidogrel:494/249 | 3630/1773 |
| TECOS | 2015 | 7332/7339 | 5743/5812 | 4647/4675 | 2976/3044 | 2444/2517 | 5764/5754  (Aspirin) | 5851/5868 |
| SAVOR-TIMI 53 | 2013 | 8280/8212 | ACEI:4435/4505  ARB:2332/2263 | 5101/5061 | — | — | 6249/6155  (Aspirin) | 6482/6435 |
| EXAMINE | 2013 | 2701/2679 | 2201/2210 | 2208/2203 | 1005/1009 | 586/611 | 2448/2433  (Aspirin) | 2420/2446 |
| VADT | 2009 | 892/899 | — | — | — | — | — | — |
| ADVANCE | 2008 | 5571/5569 | — | — | — | — | 2460/2435 | 1554/1592 |
| ACCORD | 2008 | 5128/5123 | 2718/2715 | 1472/1532 | — | — | 2810/2772  (Aspirin) | 3164/3197 |

**Supplementary Table 4. The oral anti-cardiovascular drug use before admission**
